# Kinesiotaping for postoperative oedema – what is the evidence? A systematic review

**DOI:** 10.1186/s13102-020-00162-3

**Published:** 2020-03-02

**Authors:** Julie Hörmann, Werner Vach, Marcel Jakob, Saskia Seghers, Franziska Saxer

**Affiliations:** 1 Department of Orthopaedic and Trauma Surgery, University Hospital Basel, Basel, Switzerland; 2 Crossklinik AG Swiss Olympic Medical Centre, Basel, Switzerland; 3 University of Basel, Basel, Switzerland; 4 Department of Physical Therapy, University Hospital Basel, Basel, Switzerland

**Keywords:** Kinesiotaping, Physiotape: postoperative oedema, Lymphatic drainage, Systematic review

## Abstract

**Background:**

Postoperative oedema is a common condition affecting wound healing and function. Traditionally, manual lymphatic drainage is employed to reduce swelling. Kinesiotaping might be an alternative resource-sparing approach. This article explores current evidence for the effectiveness of kinesiotaping for the reduction of oedema in the postoperative setting.

**Methods:**

A systematic literature search was performed on the basis of five databases (Pubmed, CINAHL, Embase, Cochrane Library, and Clinicaltrials.gov) for studies published between January 2000 and October 2019.

Only prospective controlled trials were included. Case studies, uncontrolled case series, studies on oedema caused by other etiologies than by surgery, as well as studies on malignant disease related oedema (especially breast cancer related oedema) were excluded.

Articles were screened by title, abstract, and full text and the references were searched for further publications on the topic. A narrative and quantitative (using STATA) analysis was performed.

**Results:**

One thousand two hundred sixty-three articles were screened, twelve were included in the analysis. All studies evaluated either oedema after extremity surgery or maxillofacial interventions, and showed relevant methodological flaws. Only three studies employed an active comparator. Of the twelve included studies ten found positive evidence for kinesiotape application for the reduction of swelling and beneficial effects on secondary outcome parameters such as pain and patient satisfaction. The available trials were heterogenic in pathology and all were compromised by a high risk of bias.

**Conclusion:**

There is some evidence for the efficacy of kinesiotaping for the treatment of postoperative oedema. This evidence is, however, not yet convincing given the limitations of the published trials. Methodologically sound comparison to standard of care or an active comparator is indispensable for an evaluation of effectiveness. In addition, assessments of patient comfort and cost-benefit analyses are necessary to evaluate the potential relevance of this novel technique in daily practice.

**Systematic review registration number:**

International prospective register of systematic reviews (PROSPERO) ID 114129).

## Background

Oedema is a pathologic condition characterized by an accumulation of fluid in the interstitium, leading to local or generalized swelling. Oedemas are differentiated in primary (a systemic and often idiopathic abnormality) and secondary oedemas (an external cause leading to venous and/or lymphatic insufficiency). Secondary oedema can be caused by a variety of reasons, such as cancer, heart failure, or trauma. Surgery is also a common cause of secondary oedema [1, 2].

Traditionally, decongestive measures, including manual lymphatic drainage and compression treatment using complex multi-layer bandaging or compression stockings, as well as skin care and decongestive exercise, have been established for the treatment of oedemas [2–4].

Recently though, kinesiotaping has gained some attention in this context. The principle was developed by the Japanese chiropractor Kenzo Kase in the seventies, and has been popularized in Europe since the nineties [2, 5, 6]. A kinesiotape is an elastic tape usually made of cotton, which contains longitudinal interwoven elastic fibers and acrylic glue that is spread in a wavelike pattern. The material has an elasticity of approximately 130–140%, and is applied to the skin using a certain amount of traction, thereby influencing the skin and various subcutaneous layers [5, 7].

Many different indications for the use of kinesiotape have been proposed, such as influencing the muscular tone, supporting joint functions, affecting pain perception, and reducing swelling [5]. Regarding the treatment of oedema, several mechanisms of action are being discussed: The pre-tension of the tape subtly lifts the skin, thereby possibly improving the lymphatic flow and directing it to pathways that suffer less congestion [5]. Furthermore, the tape is assumed to provide a massage-effect during active movement [8].

Currently there are only few individual and heterogeneous trials and there is no systematic review exploring kinesiotape application for the treatment of postoperative edema independent of malignancy. The investigation reported in this article therefore aims at evaluating the current evidence to determine the state of research and the evidence for an efficacy or effectiveness of this approach following the PICOS scheme with an analysis of **p**articipants, **i**nterventions, **c**omparisons, **o**utcomes, and **s**tudy design. This question is of relevance since superiority or even non-inferiority of kinesiotaping in the treatment of postoperative oedema might allow a change in standard management, which in turn could liberate health care professionals from resource-intensive lymphatic drainage to other important tasks like mobilization, instruction etc.

## Methods

### Types of studies

We conducted a systematic literature search to identify existing studies presenting original empirical research on the use of kinesiotape for the treatment of postoperative oedema following a predefined project plan (PROSPEROID 114129). The actual type of index-surgery was irrelevant as in- or exclusion criterion.

### Types of participants

We included prospective controlled studies published in English, German or French involving adult participants who were treated with kinesiotaping for postoperative oedema. We excluded studies analyzing the effect of kinesiotaping for oedema associated with malignancy or studies evaluating possible kinesiotape-mediated effect on muscular tonus. Equally animal studies were excluded.

### Types of interventions

Kinesiotaping for the treatment of postoperative oedema was defined as wavy application of thin kinesiotape stripes converging at lymphatic drainage centres. The type of taping was identified following the authors’ descriptions or images in the publications. Studies that stated lymphtaping but described or depicted other types of kinesiotape application were excluded. We included studies that compared kinesiotaping for the treatment of postoperative oedema to a) no specific or sham treatment, b) manual lymphatic drainage, or c) pneumatic compression.

### Types of outcome measures

Outcomes of interest were the reduction in swelling i.e. reduction in leg circumference or facial surface, pain, function, patient satisfaction and side effects, both at specific time points or with respect to the temporal course. No primary outcome was defined a priori. The plan was to analyze all outcomes reported in the majority of studies in a comparable manner.

### Search methods for identification of studies

Five databases (Pubmed, CINAHL, Embase, Cochrane Library, and Clinicaltrials.gov) were searched for published and unpublished articles. For the Cochrane Library the Cochrane Database of Systematic Reviews, the Cochrane Central Register of Controlled Trials (CENTRAL) and Cochrane Clinical Answers were searched. The search included studies that were published between January 2000 and October 2019. The exact search string for each database is reported as supporting information (S1). An overviewing search of the years 1990–1999 did not yield any publications matching the above stated inclusion criteria.

Systematic reviews on kinesiotape in general were explicitly included in the search and clearing process, in order to check for additional original articles. Also, the references of the included studies as well as the citations of these studies according to the WebOfScience were checked.

### Data collection and analysis

#### Study selection and data abstraction

Selection and data abstraction followed van Tulder et al. [9]. Two reviewers (JH and FJS) assessed the studies for eligibility screening title and abstract. Ambiguous studies were discussed in a group of three researchers (JH, FS, and WV). For articles meeting the above described inclusion criteria, full-texts were assessed for the pre-specified aspects listed in Table 1. The PICO (population, intervention, comparison, outcome) scheme was used to extract data of interest: Population characteristics comprised inclusion criteria, the average age, the gender ratio and the type of intervention. Intervention characteristics included the method of taping, the duration of treatment and the type of additional treatments equal for both groups (see below). The control intervention included active alternative treatments like lymphatic drainage or pneumatic compression, no treatment and/or sham treatment. In all studies all patients received additional supportive treatments like anti-inflammatory medication, application of cold, physiotherapy for mobilization etc. independently from their allocation to intervention- or control-group. Outcome measures included data on the course of swelling, pain levels, function, aspects of patient satisfaction and side effects. Data were extracted and documented without a specific software.

**Table 1:** Data extracted from included articles

• Journal	
• Impact factor	
• Number of patients	
• Study design	
• Drop-out rate	
• Sample size calculation	
• Patients/Population (PICO)	
• Intervention (PICO)	
• Comparison (PICO)	
• Outcome (PICO)	
• Complications	

The assessment of quality followed Higgins et al. [10] analyzing the risk of allocation bias due to randomization or allocation concealment, the risk of performance bias in the context of blinding, the risk of detection bias minimized by blinded assessment of the main outcomes, attrition bias due to incomplete outcome data and reporting bias in the context of selective reporting. The reviewers were aware of the original authors, institutions and journals for reasons of feasibility. Authors could be contacted to clarify or provide additional information if the study provided insufficient information.

### Data analysis

For a qualitative analysis, key aspects of the studies were extracted and tabulated and the main study findings were summarized verbally. For a quantitative analysis, only the degree of swelling satisfied the predefined criteria for outcome selection. Swelling was reported as (mean) circumferences/diameters (or related measures) at time points varying substantially from study to study. Many studies reported several outcome variables related to swelling without specifying a primary outcome. We hence extracted all corresponding data from all articles, aiming at computing the difference in mean values and a confidence interval at each time point reported. For eight studies, we could extract the standard deviations and sample sizes in each arm. For the study by Windisch et al. [11], we deduced standard errors from a graphical visualization of the confidence intervals of the mean values in each arm. For the study by Bialoszewski et al. [12], we made use of the *p*-values of a paired t-test comparing follow-up values with baseline values. For the study of Boguszewski et al. [13], we could not find sufficient information to compute confidence intervals. For the study by Balki et al. [14], the authors provide the mean and standard deviation values on our request.

We present the results from each study by plotting the observed difference in mean values with a 95% confidence interval at each time point. We should note that the outcomes are conceptually, but not necessarily numerically comparable. In addition, for most studies it was impossible to consider effect sizes for change scores, as the information was insufficient. Both aspects together prevent us from performing a formal meta-analysis and to assess the risk of publication bias.

### Registration

The review was registered with PROSPERO (ID 114129).

## Results

**Fig. 1:**
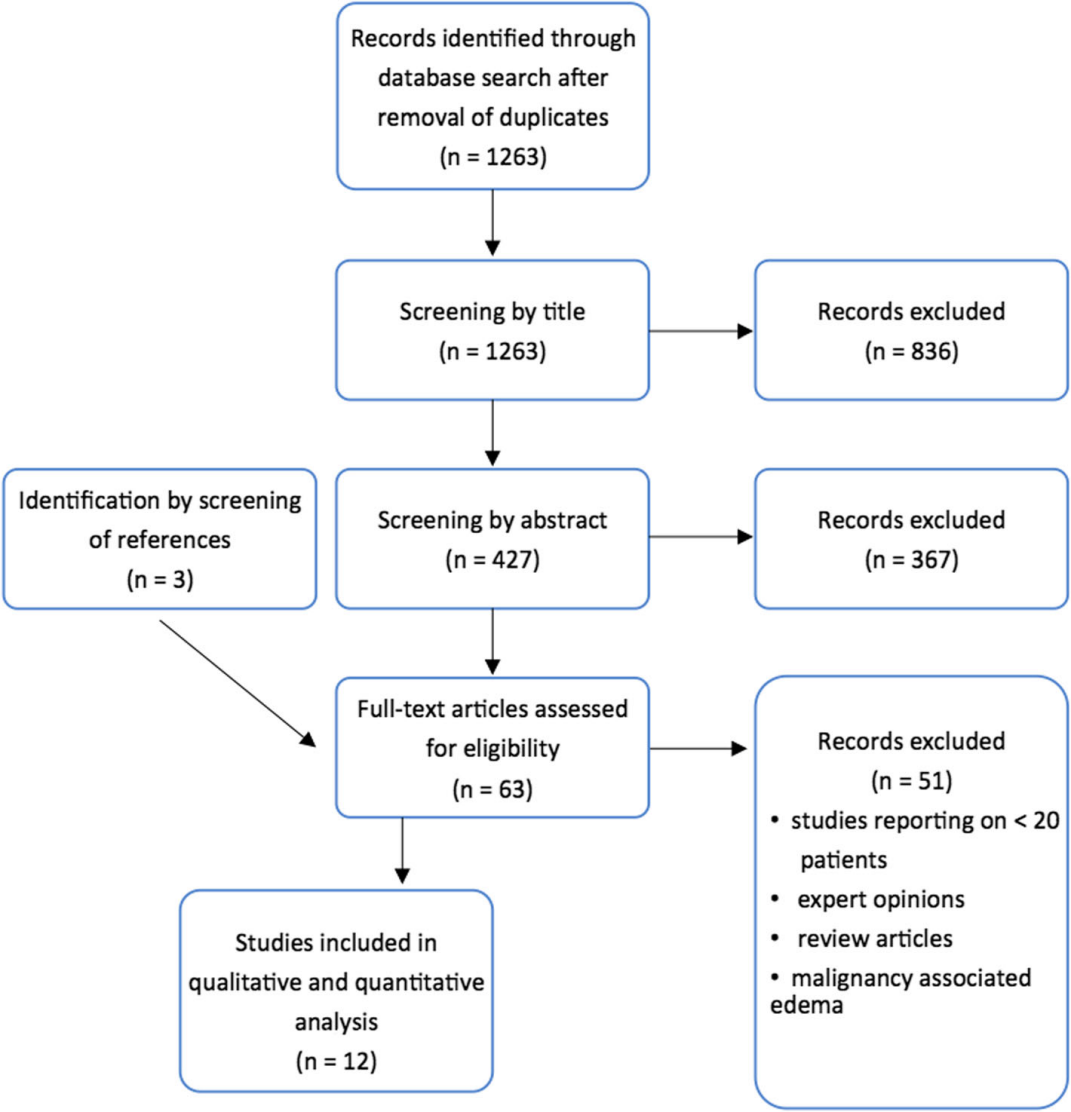
Flow Chart for article selection

A total of 1263 articles were identified by our search strategy after removal of duplicates. These were screened by title, abstract, and, if potentially qualifying, by full text. We identified ten studies for analysis. Both the references within these publications and the citations of these studies allowed identifying three further studies. Finally, twelve studies were consistent with the pre-defined criteria. A flow diagram of the screening process is presented in Fig. 1. No previous systematic review considering kinesiotape as a treatment for postoperative oedema etiologically independent of malignancy could be identified.

### Qualitative analysis of included studies

Eleven articles described prospective randomized controlled trials (RCTs), and one article described a prospective case series with a historic control. Table 2 shows a comparative overview of key aspects. A qualitative description of the included studies is presented in the supplemental material as supporting information (S2).

**Table 2:** List of eligible publications and key features

Authors	Research methodology	Population/Patients	Surgical intervention	Area of application	Intervention	Comparison/Control	Outcomes	Follow up	Drop out Rate	Conclusion for reduction of edema
Bialoszewski et al. [12] 2009	RCT single center	24 patients age 15–46 years	Leg lengthening with Ilizarov approach	Tight and crus	kinesiotaping in addition to control treatment, picture documentation	**- manual lymphatic drainage**	- Limb circumference	approx. 10 day	0%	Leg circumference _0_: ➢ Significant in 5/6 locations K-Tape ➢ Significant in 3/3 locations control **→ favours K-Tape**
Boguszewski et al. [13] 2013	RCT single center	26 patients age 20–41 years	ACL reconstruction	Knee	kinesiotaping in addition to control treatment, detailed description	- isometric exercise - non-weight-bearing active exercises - self-assisted exercises in closed and open kinetic chains - proprioceptive exercises - stationary bike workout	- ROM - Limb circumference - Musculoskeletal pain - Perceived effect of physiotherapy	4 weeks	0%	Leg circumference at knee level _0_: ➢ High levels of significance at early time points K-Tape ➢ Low levels of significance at early time points control **→ favours K-Tape**
Balki et al. [14] 2016	RCT single center	30 patients age 18–39 years; mean age 28.1 years	ACL reconstruction	Knee	Kinesiotaping and physiotherapy	**- Sham taping** **-** physiotherapy	- Pain - Swelling - ROM - muscular strenght		0%	Leg circumference _0_: ➢ Significant difference midpatellar day 5, in 3/3 locations day 10 postop. **→ favours K-Tape**
Chan et al. [15] 2017	RCT single center	60 patients average age 26.85 years	ACL reconstruction	Knee	kinesiotaping in addition to control treatment, detailed description	- soft tissue mobilization - joint mobilization - gait retraining - therapeutic exercise - electrical physical modalities	- Pain score - Lysholm–Tegner Score - Mid Patellar Girth - ROM	6 weeks	0%	Leg circumference at knee level _0_: ➢ No significant difference at early or late time points **→ no favour**
Donec et al. [16] 2014	RCT single center	89 patients average age 67.35 years	primary total knee replacement surgery	Knee	kinesiotaping in addition to control treatment, detailed description and picture documentation	**- intermittent pneumatic compression** - physiotherapy - early mobilization - occupational therapy - massage - TENS - laser therapy - paraffin therapy - psychologist and social work care	- Pain score - Reduction of edema - ROM	28 days	5%	Leg circumference at the level of the tight, knee and calf_0_: ➢ Significant differences at early postoperative time points Leg circumference at the level of the ankle joint _0_: ➢ No significant differences between treatment groups **→ favours K-Tape**
Windisch et al. [11] 2017	Prospective with historical control single center	42 patients age range 47–86 years	Total knee replacement	Knee	kinesiotaping (detailed description and picture documentation) instead of AV Impulse System™	**- AV Impulse System™ 24 h** unless during active physiotherapy and ADL training - physiotherapeutic regime including continuous passive motion and active treatment - training activities of daily living (ADL)	- Duration of postoperative wound secretion - Leg circumference - thermographic temperature determination	7 days	0%	Leg circumference _0_: ➢ no significant difference at any time or measuring point **→ no favour**
Gülenç et al. [17]2018	RCT single center	42 patients, older than 18 years, mean age control group: 42.25 years mean age intervention group: 40.6 years	Knee arthroscopy	Knee	Kinesiotaping, detailed description and picture documentation	**Sham taping**	- Pain score- Limb diameter	6 weeks	16%	Limb circumference at the level of the thigh and ankle: ➢ No significant difference at early or late time points Limb circumference at the knee level: ➢ Significant difference at early and late time points Limb circumference at calf level: ➢ Significant difference at late time points **→ favours K-Tape**
Gülenç et al. [18]2019	RCT single center	58 patients, 18–50 years	Shoulder arthroscopy	Shoulder	Kinesiotaping, detailed description and picture documentation	**Sham taping**, detailed description and picture documentation	- Pain score- Shoulder diameter	6 weeks	14%	Upper shoulder diameter: ➢ No significant difference at early or late time points Lower shoulder diameter: ➢ Significant difference during follow up, but not on first or last measurement **→ favours K-Tape**
Ristow et al. [19] 2013	RCT single center	26 patients age range 18–75 years	ORIF of unilateral mandibular fractures	Head/Neck	kinesiotaping in addition to control treatment, detailed description and picture documentation	- cooling - analgesia - antibiotic treatment	- Extent of max. Swelling - Extent of swelling on postoperative days 1–3 - Time of maximal swelling - Extent of detumescence within 1d of max. Swelling - Interincisal distance - Pain - Subjective outcomes on tape comfort - Movement limitation through tape - Subjective sensation of swelling - Patient satisfaction	7 days	0%	Face surface (sum of measurement lines) _1_: ➢ Non-significant differences from max. Swelling to the day after ➢ Significant differences for increase of swelling **→ favours K-Tape**
Ristow et al. [16] 2014a	RCT single center	40 patients average age 27 years	Removal of bilateral upper and lower wisdom teeth	Head/Neck	kinesiotaping in addition to control treatment, detailed description and picture documentation	- cooling - analgesia	- Change in facial surface between day 0 and day 2 - Extent of max. Swelling - Time of maximal swelling - Extent of detumescence within 1d of max. Swelling - Pain - Mouth opening - Subjective outcomes on tape comfort - Movement limitation through tape - Subjective sensation of swelling - Patient satisfaction	7 days	0%	Face surface (sum of measurement lines) _1_: ➢ Significant differences from max. Swelling to the day after ➢ Significant differences for increase of swelling **→ favours K-Tape**
Ristow et al. [20] 2014b	RCT single center	30 patients age range 18–74 years	ORIF of zygomatico-orbital/ zygomatic-maxillary fractures involving the orbital floor	Head/Neck	kinesiotaping in addition to control treatment, detailed description and picture documentation	- cooling - analgesia	- Increase of swelling - Extent of maximal swelling - Time of maximal swelling - Extent of detumescence within 1d of max. Swelling - Pain - Mouth opening - Subjective outcomes on tape comfort - Movement limitation through tape - Subjective sensation of swelling - Patient satisfaction	7 days	0%	Face surface (sum of measurement lines) _1_: ➢ Non-significant differences from max. Swelling to the day after ➢ Significant differences for increase of swelling **→ favours K-Tape**
Tozzi et al. [21] 2016	RCT single center	24 patients age range 18–37 years	Bimaxillary orthognathic surgery	Head/Neck	kinesiotaping in addition to control treatment, detailed description and picture documentation	- perioperative steroids	- Change in facial surface between day 0 and day 2 - Pain - Mouth opening	4 days	0%	Face surface (3D molding) _1_: ➢ Significant differences for increase of swelling **→ favours K-Tape**

### Quantitative analysis

**Fig. 2:**
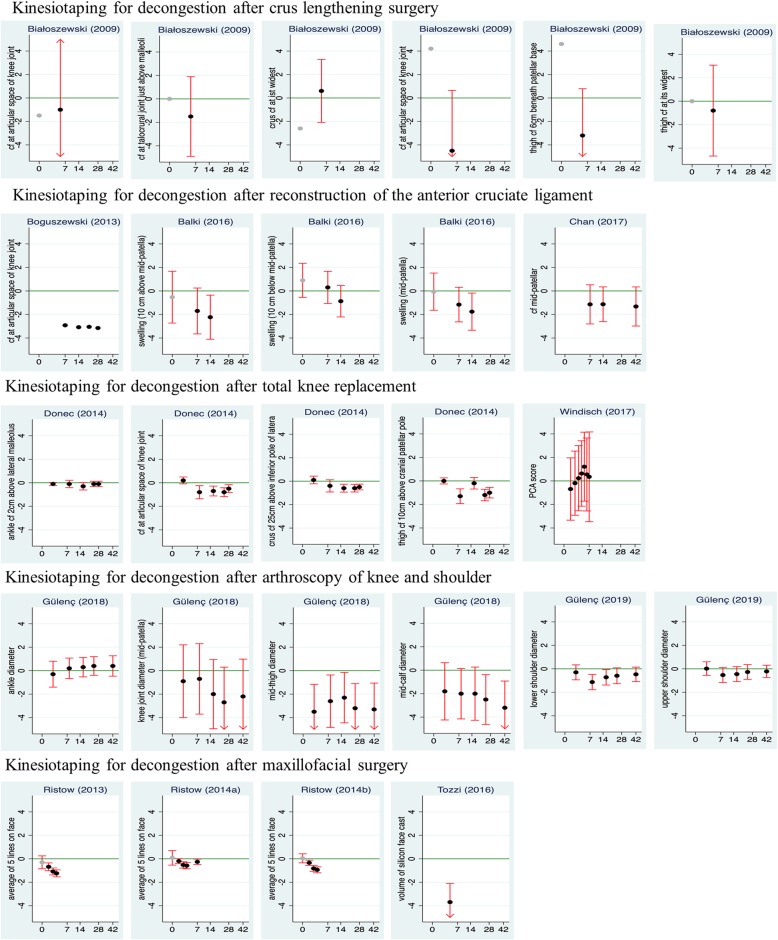
Differences in Swelling. Difference in mean values (black points) between the kinesiotape group and the control group for all outcome variables directly assessing the degree of swelling and for all time points reported in the studies. Negative differences indicate better outcomes under kinesiotape. In the studies of Donec et al. [22] and Ristow et al. [16, 19, 20] and for day 5 in the study of Bialoszewski et al. [12] results are based on change scores, in all other studies raw measurements are used as input. Most studies report a circumfence or diameter as outcome. For the study by Windisch et al. [11] we use the score from a “Principal Component Analysis2 based on eight different circumferences and omitted the eight single outcomes. For the maxillo-facial evaluaions, the three studies by Ristow et al. [16, 19, 20] use the sum of five predefined line lengths in the face, the study of Tozzi et al. [21] a volume based on a MakerBot® Digitizer 3DTM in cm^3^.95% confidence intervals (red lines) are shown when sufficient information was provided in the studies. They are truncated at − 5 or 5, as indicated by arrows. The green line refers to no difference between the two groups. The x-axis refers to time in days and is square root transformed. Results for differences at baseline are marked in gray. Studies are indicated by the name of the first author and the year of the publication cf.: circumference.

The only quantitative outcomes that were assessed in a conceptually comparable way across the majority of studies were the extent of swelling and pain. Since the choice of pain scales and numerical reporting practice for pain varied considerably, only the degree of swelling qualified as criterion for a quantitative analysis in all twelve studies. Figure 2 presents differences in mean values between the intervention groups and the control groups for the outcome variables related to swelling from all studies.

Over all studies and all outcome variables, we observe a majority of negative differences when excluding very early assessments. This means less swelling with additional kinesiotape treatment compared to control treatment only. The only distinct exception is the study by Windisch et al. [11]. Four studies provide rather clear statistical evidence for an advantage of kinesiotaping: the study of Tozzi et al. [21] considering a single outcome, and the studies by Ristow et al. [16, 19, 20] which indicate an increasing difference over time, reaching significance at day 2 the latest. Also in the study by Donec et al. [22], we can recognize significant differences concerning three of the four outcomes at several follow-up time points, in the study of Gülenc [17] for two of four outcomes at several time points, and in the study of Balki et al. [14] for two outcomes on day 10.

### Side effects

Five studies stated no adverse effects of taping; two studies reported of one (1/25 [17],) and two (2/35 [15],) patients respectively having had a skin reaction that lead to an interruption in treatment. The other studies did not comment on kinesiotape related complications.

## Discussion

### Summary of results

We could identify 12 studies comparing kinesiotaping for the management of postoperative oedema to other management options in a variety of patient populations. Eleven of these studies were RCTs. Estimates of the difference in swelling between the treatment groups suggested a beneficial effect of kinesiotape in many studies. However, the statistical significance of the findings in the single studies was varying and remained often unclear. It was not possible to conduct a formal meta-analysis, as the swelling was measured at different body parts and by different techniques. Furthermore, all studies were affected by a high risk of bias. Another recent trial has not yet been published but results from a conference abstract imply a significant reduction of pain and oedema after both kinesiotaping and MLD compared to control after total knee replacement [23]. The trial could not be included in the review since detailed data were not available upon request from the author.

An evaluation of the effectiveness was hampered be the fact that only three studies [11, 13, 22] involved an active comparator, two of them a pneumatic compression system and one manual lymphatic drainage. The study by Bialoszewski et al. [12] as the only one comparing kinesiotaping to manual lymphatic drainage as current gold standard suffers from methodologic flaws and lacks a description of the patient population or a comparison of the two groups. Also, our quantitative analysis of this study indicates no clear treatment effect. This leaves the studies by Donec et al. [22] and Windisch et al. [11] that share a similar patient population and active comparator. Unfortunately, their conclusions are conflicting. Hence, the evidence on which to base the recommendation of kinesiotaping for the treatment of postoperative oedema is rather limited.

### Risk of bias

**Fig. 3:**
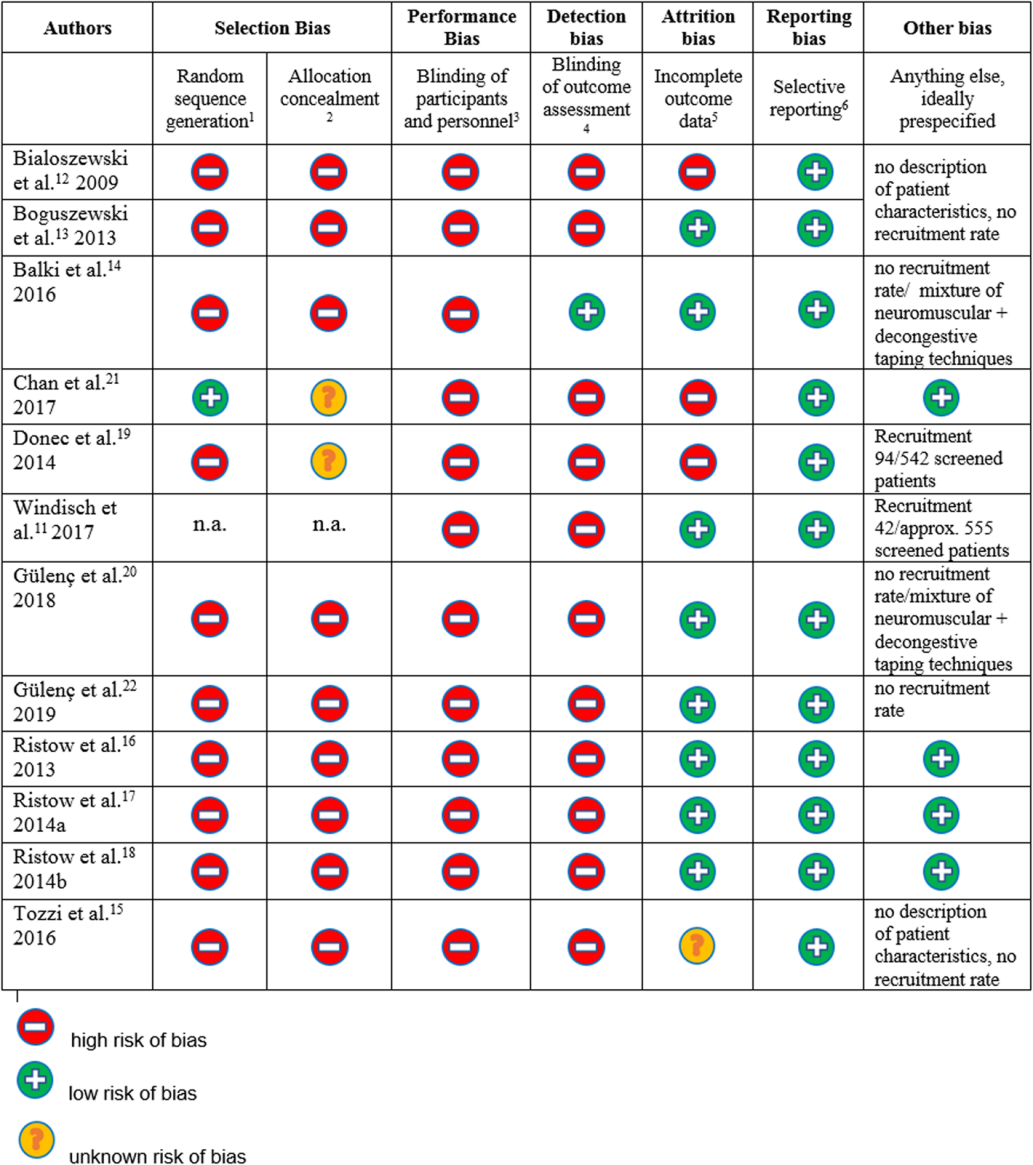
Risk of bias assessment. ^1^ random sequence generation: none of the articles described random sequence generation in detail. The study by Bialoszewski et al. [12] is affected by an even higher risk since patients were not randomized primarily but only if they developed oedema during treatment. Chan et al. [21] recruited patients with and without meniscal surgery which might be medically reasonable but is methodologically disputable. Windisch et al. [11] performed no randomization but used a historical control. Gulenc et al. [17] describe randomization “ based on the rank of admission” in their study on kinesiotaping after knee arthroscopy. ^2^ Allocation concealment is not described or doubtful (picking of envelopes) ^3^ Blinding of participants and personnel is not feasible in this context since the effect of sham taping with an alternative material has not been explored and control treatment like manual lymphatic drainage or intermittent pneumatic compression cannot be concealed either. ^4^ All but one articles fail to mention a blinding of the assessor, only Donec et al. [22], Ristow et al. [16, 19, 20] and Tozzi et al. [21] name the assessor. Balki et al. [14] describe a separation of assessor and researcher. ^5^ Bialoszewski et al. [12] miss to report the exact duration of treatment as well as the exact timing of assessment, Chan et al. do not mention the exact timing of assessments. Donec et al. [22] fail the reporting of basic measurements preoperative and retrospectively retrieve data on use of analgesics from patients’ charts. Tozzi et al. [21] do not report the beginning of treatment. ^6^ In spite of the overall high risk of bias in all the studies a tendency for selective reporting cannot be observed.

The risk of bias is displayed in Fig. 3 as proposed by Higgins et al. [10]. Performance bias cannot be excluded, as none of the studies used an adequate sham–taping as control, hence blinding of participants and personnel was impossible. Balki et al. [14] describe sham taping with a broad strip of non-tensioned kinesiotape on the anterior and posterior distal thigh. An adequate sham-control though should visually imitate the treatment under investigation without exerting its potential effect. The studies by Gülenç et al. [17, 18] did compare kinesiotaping to a sham-taping that indeed seems to have mimicked the application technique (at least in the area of the shoulder [18], no further information has been available in the article or after contacting the author on the sham-taping around the knee), but used a tape clearly different from kinesiotape by texture and appearance [18].

All studies failed to report blinding of the assessor. Only one accurately described the random sequence generation, and none choose more reliable randomization tools than sealed envelopes. Only four studies specified a primary outcome.

Besides these threads to the internal validity of the study, the external validity was also questionable: only one study used the current standard of care (manual lymphatic drainage) as comparator, only another two used an alternative active comparator. The information on the patient population was insufficient in all studies. The comparability of the studies additionally suffered from the variation in outcome measures and in follow-up time points.

### The broader context

When interpreting the results, studies from maxillofacial surgery and extremity surgery should be separated. Ristow et al. [16, 19, 20] describe a standardized postoperative regimen with non-steroidal antiphlogistic medication as analgesic medication with influence though on inflammation and swelling, as well as and application of cooling measures. Tozzi et al. [21] used the application of perioperative dexamethasone and cooling as antiphlogistic treatment. Analgesic treatment is not reported by Tozzi et al. [21], seems probable though with potential influence on oedema development and resorption. Manual lymphatic drainage for the treatment of oedema after maxillofacial surgery does not seem as popular as in other fields of surgery. There are, however, publications that could show its benefit [24–26], and one ongoing trial is evaluating its clinical relevance [27]. Two studies – not included in this review due to the lack of control group in one and the lack of detailed information from a conference abstract in the other– state a benefit of kinesiotape application after penile surgery [28, 29], emphasizing the advantageous versatility of the technique that is adaptable to various anatomic regions. One additional study that lacked a control group and was therefore equally excluded in this review concludes a benefit of kinesiotaping after orthognatic surgery for the reduction of postoperative swelling [30].

**Fig. 4:**
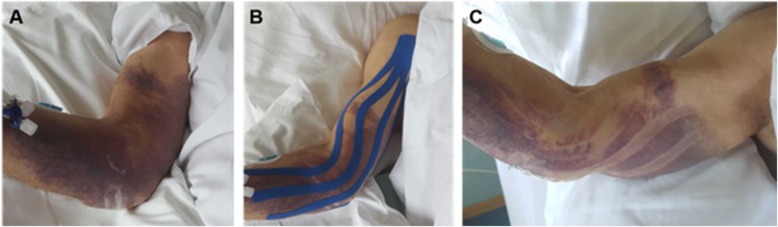
Kinesiotape application. Clinical effect of kinesiotape application in an elderly patient with an extensive hematoma of the right upper extremity (**a**). After kinesiotape application (**b**) and removal (**c**) signs of resorption can be noted at the former location of kinesiotape

Considering extremity surgery, manual lymphatic drainage is broadly accepted for the treatment of postoperative and posttraumatic oedema [31–33] as well as oedema caused by other pathologies [2, 29, 30], even though corresponding evidence is conflicting [3, 4, 34, 35]. The application of pneumatic compression was also established as treatment option [36–38], although again with limited evidence base [38–41]. Kinesiotaping might be yet another approach for the treatment of oedema. Animal experiments have shown effects on the development of oedema, the dermal structure [7], and lymphatic flow [8]. Indeed, the morphology of hematoma after application of kinesiotape (see Fig. 4) implies some effect. Whether this effect is of clinical relevance compared to other treatment modalities, the optimal technique, and treatment duration remains, however, unclear.

The treatment of oedema remains an important aspect of postoperative therapeutic regimen, especially since oedema can negatively impact function and wellbeing. In addition, oedema have been found to be associated with prolonged wound healing and infections [42–44].

Given the high costs for personnel and the durability of up to 5 days of kinesiotaping, kinesiotaping is an inexpensive form of treatment compared to manual lymphatic drainage. It seems to be well accepted by most patients, and its application probably has benefits for the patient. Skin reactions are well possible, as also reported for three patients (of > 200 patients in all trials treated with kinseiotape) in our investigation., In general, kinesiotaping might be considered an alternative treatment of postoperative oedema which optimizes resources without jeopardizing the patients’ recovery.

### Future research

There is an obvious need for more trials in well-defined patient-populations, covering specific indications and treatment aspects (ROM (range of motion), oedema, muscle strength, pain, etc.) while minimizing the risk of bias. Active comparators should be chosen that reflect the current standard, and a primary outcome directly related to swelling (or respective pathologies) should be predefined. Swelling is well suited as primary outcome, as it is relevant for the patient due to causing discomfort or even pain and simultaneously reflects the clinical target of the intervention. All studies included in our review suggest that an effect is visible after 7 days and does not increase later, suggesting 7 days as reasonable follow-up time point. Secondary outcome variables like pain, function, and wound-healing should also be addressed systematically and not least the cost-benefit ratio. In addition, a later time point might be chosen for an assessment of the clinical outcome via patient reported outcome measures (PROMS), occurrence of complications, and return to previous activities of daily living.

## Conclusions

In conclusion, there are many RCTs suggesting a positive effect of kinesiotape application on postoperative swelling in a variety of indications. There is today, however, a lack of solid evidence with respect to its effectiveness that could support a recommendation of this practice. Larger randomized controlled trials for each specific indication will be necessary for the generation of solid evidence. Kinesiotape could have a relevant impact on clinical practice and health care expenditure if indeed a similar efficacy compared to MLD as current standard of care could be demonstrated.

## Supplementary information

**Additional file 1.** S1 File: Search strings. Search strings for the following databases: - Pubmed. - CINAHL. – Embase. - Cochrane Library. • Cochrane Database of Systematic Reviews. • Cochrane Central Register of Controlled Trials (CENTRAL). •Cochrane Clinical Answers. - Clinicaltrials.gov. S2 File: Detailed qualitative description

## Data Availability

Data and additional are available upon request to the corresponding author.

## Abbreviations

CINAHL: Cumulative Index to Nursing and Allied Health Literature
ID: Identificator
MeSH: Medical Subject Headings
PICOS: Participants, interventions, comparisons, outcomes, study design
PROM: Patient Reported Outcome Measure
PROSPERO: International prospective register of systematic reviews
RCT: Randomized controlled trial
ROM: Range of motion

## References

[1] Scallan J, Huxley V, Korthuis R (2010). Pathophysiology of edema formation. Capillary Fluid Exchange: Regulation, Functions, and Pathology.

[2] Bringezu G, Schreiner O. Lehrbuch der Entstauungstherapie. Grundlagen, Beschreibung und Bewertung der Verfahren, Behandlungskonzepte für diePraxis. 4., vollständig überarbeitete und erweiterte Auflage. Berlin: Springer Medizin Verlag GmbH; 2014.

[3] Preston NJ, Seers K, Mortimer PS. Physical therapies for reducing and controlling lymphoedema of the limbs. Cochrane Database Syst Rev. 2004;(4). 10.1002/14651858.CD003141.pub2.10.1002/14651858.CD003141.pub215495042

[4] Ezzo J, Manheimer E, McNeely ML, Howell DM, Weiss R, Johansson KI, et al. Manual lymphatic drainage for lymphedema following breast cancer treatment. 2015;5:CD003475. 10.1002/14651858.CD003475.pub2 PMC4966288 25994425

[5] Kase K, Wallis J, Kase T (2003). Clinical therapeutic applications of the kinesio taping method.

[6] Delaire M (2014). Les bandages adhésifs de couleur: un nouveau concept. Kinesitherapie.

[7] Kafa N, Citaker S, Omeroglu S, Peker T, Coskun N, Diker S (2015). Effects of kinesiologic taping on epidermal-dermal distance, pain, edema and inflammation after experimentally induced soft tissue trauma. Physiother Theory Pract.

[8] Shim JY, Lee HR, Lee DC (2003). The use of elastic adhesive tape to promote lymphatic flow in the rabbit hind leg. Yonsei Med J.

[9] Van Tulder M, Furlan A, Bombardier C, Bouter L (2003). Updated method guidelines for systematic reviews in the Cochrane collaboration back review group. Spine.

[10] Higgins JPT, Altman DG, Gøtzsche PC, Jüni P, Moher D, Oxman AD (2011). The Cochrane Collaboration’s tool for assessing risk of bias in randomised trials. BMJ..

[11] Windisch C, Brodt S, Roehner E, Matziolis G, C. W, S. B (2017). Effects of Kinesio taping compared to arterio-venous impulse system (TM) on limb swelling and skin temperature after total knee arthroplasty. Int Orthop.

[12] Bialoszewski D, B WW, Sawomir B (2009). Clinical efficacy of kinesiology taping in reducing edema of the lower limbs in patients treated with the Ilizarov method – preliminary report is c op y is for pe rs on us e o nly - d istr ibu Th tio n p roh ibit Przydatnoœæ kliniczna metody. Kinesiology T.

[13] Boguszewski D, Tomaszewska I, Adamczyk JG, Bialoszewski D (2013). Evaluation of effectiveness of kinesiology taping as an adjunct to rehabilitation following anterior cruciate ligament reconstruction. Preliminary report. Ortop Traumatol Rehabil.

[14] Balki S, Göktaş HE (2016). öztemur Z. Kinesio taping as a treatment method in the acute phase of ACL reconstruction: a double-blind, placebo-controlled study. Acta Orthop Traumatol Turc.

[15] Chan MC-E, Wee JW-J, Lim M-H (2017). Does kinesiology taping improve the early postoperative outcomes in anterior cruciate ligament reconstruction? A randomized controlled study. Clin J Sport Med.

[16] Ristow O, Hohlweg-Majert B, Stürzenbaum SR, Kehl V, Koerdt S, Hahnefeld L (2014). Therapeutic elastic tape reduces morbidity after wisdom teeth removal-a clinical trial. Clin Oral Investig.

[17] Gülenc B, Kuyucu E, Bicer H, Genc SG, Yalcin S, Erdil M (2018). Kinesiotaping reduces knee diameter but has no effect on differences pain and edema following knee artroscopy. Acta Chir Orthop Traumatol Cech.

[18] Gülenç B, Yalçin S, Genç SG, Biçer H, Erdil M (2019). Is kinesiotherapy effective in relieving pain and reducing swelling after shoulder arthroscopy?. Acta Chir Orthop Traumatol Cech.

[19] Ristow O, Hohlweg-Majert B, Kehl V, Koerdt S, Hahnefeld L, Pautke C (2013). Does elastic therapeutic tape reduce postoperative swelling, pain, and trismus after open reduction and internal fixation of mandibular fractures?. J Oral Maxillofac Surg.

[20] Ristow O, Pautke C, Kehl V, Koerdt S, Schwärzler K, Hahnefeld L (2014). Influence of kinesiologic tape on postoperative swelling, pain and trismus after zygomatico-orbital fractures. J Cranio-Maxillofacial Surg.

[21] Tozzi U, Santagata M, Sellitto A, Tartaro GP (2016). Influence of Kinesiologic tape on post-operative swelling after orthognathic surgery. J Maxillofac Oral Surg.

[22] Donec V, Krisciunas A, Donec VKA (2014). The effectiveness of Kinesio taping® after total knee replacement in early postoperative rehabilitation period. A randomized controlled trial. Eur J Phys Rehabil Med.

[23] Guney Deniz H, Kinikli GI, Onal S, Sevinc C, Caglar O, Yuksel I (2018). THU0727-HPR comparison of kinesio tape application and manual lymphatic drainage on lower extremity oedema and functions after total knee arthroplasty. Ann Rheum Dis.

[24] Yaedú RYF, Mello MDAB, Tucunduva RA, Da Silveira JSZ, Takahashi MPMS, Valente ACB (2017). Postoperative orthognathic surgery edema assessment with and without manual lymphatic drainage. J Craniofac Surg.

[25] Szolnoky G, Szendi-Horváth K, Seres L, Boda K, Kemény L (2007). Manual lymph drainage efficiently reduces postoperative facial swelling and discomfort after removal of impacted third molars. Lymphology.

[26] Pavlov NV, Pechalova PF (2016). Manual lymphatic drainage techniques reduces postoperative facial swelling after third molar surgery. Arta Medica.

[27] Impact of Manual Lymphatic Drainage on Postoperative Edema of the Face and the Neck After Orthognathic Surgery (DLMOF). ClinicalTrials.gov Identifier: NCT01983436. Available from https://clinicaltrials.gov/ct2/show/NCT01983436.

[28] Bittner L, Zámečník L. Inflatable penile prosthesis implantation with scrotal kinesiology taping — novel approach to postoperative scrotal swelling prevention Eur Urol Suppl. 2017;16(11);e2920.

[29] Bittner L, Zámečník L, Valka R, Bettocchi C (2019). HP-08-004 kinesiology taping of scrotum- an update of “mummy wrap”. J Sex Med.

[30] Lietz-Kijak D, Kijak E, Krajczy M, Bogacz K, Łuniewski J, Szczegielniak J (2018). The impact of the use of kinesio taping method on the reduction of swelling in patients after orthognathic surgery: a pilot study. Med Sci Monit.

[31] Vairo GL, Miller SJ, McBrier NM, Buckley WE (2009). Systematic review of efficacy for manual lymphatic drainage techniques in sports medicine and rehabilitation: an evidence-based practice approach. J Man Manip Ther.

[32] Majewski-Schrage T, Snyder K (2016). The effectiveness of manual lymphatic drainage in patients with orthopedic injuries. J Sport Rehabil.

[33] Ebert JR, Joss B, Jardine B, Wood DJ (2013). Randomized trial investigating the efficacy of manual lymphatic drainage to improve early outcome after total knee arthroplasty. Arch Phys Med Rehabil.

[34] Finnane A, Janda M, Hayes SC (2015). Review of the evidence of lymphedema treatment effect. Am J Phys Med Rehabil.

[35] Stuiver MM, ten Tusscher MR, Agasi-Idenburg CS, Lucas C, Aaronson NK, Bossuyt PMM (2015). Conservative interventions for preventing clinically detectable upper-limb lymphoedema in patients who are at risk of developing lymphoedema after breast cancer therapy. Cochrane Database Syst Rev.

[36] Zaleska M, Olszewski WL, Durlik M (2014). The effectiveness of intermittent pneumatic compression in long-term therapy of lymphedema of lower limbs. Lymphat Res Biol.

[37] Windisch C, Kolb W, Kolb K, Grützner P, Venbrocks R, Anders J (2011). Pneumatic compression with foot pumps facilitates early postoperative mobilisation in total knee arthroplasty. Int Orthop.

[38] Clarkson R, Mahmoud SSS, Rangan A, Eardley W, Baker P (2017). The use of foot pumps compression devices in the perioperative management of ankle fractures: systematic review of the current literature. Foot..

[39] Tran K, Argáez C. Intermittent Pneumatic Compression Devices for the Management of Lymphedema: A Review of Clinical Effectiveness and Guidelines. Ottawa: CADTH; (CADTH rapid response report: summary with critical appraisal). 2017. 29553689

[40] Stout N, Partsch H, Szolnoky G, Forner-Cordero I, Mosti G, Mortimer P (2012). Chronic edema of the lower extremities: international consensus recommendations for compression therapy clinical research trials. Int Angiol.

[41] Winge R, Bayer L, Gottlieb H, Ryge C (2017). Compression therapy after ankle fracture surgery: a systematic review. Eur J Trauma Emerg Surg.

[42] Baddour LM, Bisno AL (1985). Non-group a beta-hemolytic streptococcal cellulitis. Association with venous and lymphatic compromise. Am J Med.

[43] Myers M, Cherry G, Heimburger S, Hay M, Haydel H, Cooley L (1967). The effect of edema and external pressure on wound healing. Arch Surg.

[44] Itobi E, Stroud M, Elia M (2006). Impact of oedema on recovery after major abdominal surgery and potential value of multifrequency bioimpedance measurements. Br J Surg.

