# Supplementary material for: Kinesiotaping for postoperative oedema – what is the evidence? A systematic review
Source: BMC Sports Sci Med Rehabil. 2020 Mar 2;12:14. doi: 10.1186/s13102-020-00162-3 (PMC7052984; doi:10.1186/s13102-020-00162-3)
Supplement: Supplementary file 1 — Additional file 1. S1 File: Search strings. Search strings for the following databases: - Pubmed. - CINAHL. – Embase. - Cochrane Library. • Cochrane Database of Systematic Reviews. • Cochrane Central Register of Controlled Trials (CENTRAL). •Cochrane Clinical Answers. - Clinicaltrials.gov. S2 File: Detailed qualitative description [file 13102_2020_162_MOESM1_ESM.docx]

# Supplemental material

Search string Pubmed:

((((lymphedem*[Text Word] OR Lymphoedem*[Text Word] OR Lymphodem*[Text Word] OR edem*[Text Word] OR oedem*[Text Word] OR odem*[Text Word] OR lymphedema[MeSH Terms] OR edema[MeSH Terms])) AND (tape[All Fields] OR tapes[All Fields] OR taping[All Fields] OR kinesiotap*[All Fields] OR kinesio-tap*[All Fields] OR kinesio-tex*[All Fields] OR Kinesio Tex[All Fields] OR K-tap*[All Fields] OR K tap*[All Fields] OR kinesio tap*[All Fields] OR kinesiology tap*[All Fields] OR strapping[All Fields] OR kinesio[All Fields] OR athletic tape[MeSH Terms] OR surgical tape[MeSH Terms]))) OR Lymphtap*[All Fields]

Search string CINAHL:

Search 1:

lymphedem* OR Lymphoedem* OR Lymphodem* OR edem* OR oedem* OR odem* OR (MH lymphedema+) OR (MH edema+)

Search 2:

tape OR tapes OR taping OR kinesiotap* OR kinesio-tap* OR kinesio-tex* OR Kinesio Tex OR K-tap* OR K tap* OR kinesio tap* OR kinesiology tap* OR strapping OR kinesio OR (MH athletic tape+) OR (MH tapes+)

Search 3:

Lymphtap*

Search 1 AND Search 2 = Search 4

Search 4 OR Search 3 = Search 5 = Final Search

Search string Embase:

Search 1:

'lymphedem*':ti,ab,kw OR 'lymphoedem*':ti,ab,kw OR 'lymphodem*':ti,ab,kw OR 'edem*':ti,ab,kw OR 'oedem*':ti,ab,kw OR 'odem*':ti,ab,kw OR 'ankle edema'/exp OR 'arm edema'/exp OR 'foot edema'/exp OR 'generalized edema'/exp OR 'hand edema'/exp OR 'leg edema'/exp OR 'peripheral edema'/exp OR 'postoperative edema'/exp OR 'lymphedema'/exp

Search 2:

'tape':ti,ab,kw OR 'tapes':ti,ab,kw OR 'taping':ti,ab,kw OR 'kinesiotap*':ti,ab,kw OR 'kinesio-tap*':ti,ab,kw OR 'kinesio-tex*':ti,ab,kw OR 'kinesio tex':ti,ab,kw OR 'k-tap*':ti,ab,kw OR 'k tap*':ti,ab,kw OR 'kinesio tap*':ti,ab,kw OR 'kinesiology tap*':ti,ab,kw OR 'strapping':ti,ab,kw OR 'kinesio':ti,ab,kw OR 'kinesiotherapy'/exp OR 'kinesio taping'/exp OR 'kinesio tape'/exp OR 'kinesiotaping'/exp OR 'athletic tape'/exp OR 'surgical tape'/exp

Search 3:

'lymphtaping':ti,ab,kw

Search 1 AND Search 2 = Search 4

Search 4 OR Search 3 = Search 5 = Final Search

Search string Cochrane Library:

Search 1:

lymphedem* OR Lymphoedem* OR Lymphodem* OR edem* OR oedem* OR odem* OR [mh edema] OR [mh lymphedema]

Search 2:

tape OR tapes OR taping OR kinesiotap* OR kinesio-tap* OR kinesio-tex* OR Kinesio Tex OR K-tap* OR K tap* OR kinesio tap* OR kinesiology tap* OR strapping OR kinesio OR [mh “athletic tape”] OR [mh “surgical tape”]

Search 3:

Lymphtap*

Search 1 AND Search 2 = Search 4

Search 4 OR Search 3 = Search 5 = Final Search

Search string Clinical trials gov:

Lymphedema and tape

Lymphedema and Kinesio

**Detailed qualitative description**

**Extremity Surgery**

The study by Bialoszewski et al.[12] included two groups of each 12 patients undergoing crus or thigh lengthening using the Ilizarov method[46,47] and compared kinesiotape as addition to manual lymphatic drainage (MLD) to manual drainage only in patients developing edema after surgery. However, the description remains vague concerning the taping technique, the duration of treatment, and measurement time points. In both groups, multiple measurements were performed at three levels of the extremity shortly after surgery and at later time points. Regarding the primary endpoint reduction of edema during the course of treatment, the study found a positive effect in tight lengthening at all three levels of measurement in the kiensiotape group, but only two in the control group. For patients with crus lengthening, a reduction of edema was reached at two levels of measurement compared to only one in the control group. No intergroup comparison was performed and no other outcomes were assessed or reported. The authors conclude a relevant effect of both Kinesiotaping and MLD for postoperative edema, with a more rapid action after the application of kinesiotape.

Boguszewski et al.[13] investigated the effectiveness of kinesiotape on total 26 patients after ACL reconstruction. All patients were treated with standardized physiotherapy, and the intervention group additionally with kinesiotape. Exact information about the beginning of treatment is lacking. Kinesiotaping was performed for 4 weeks, with change of tape every 7 days by means of a mixed lymph- and muscle-activating technique. The authors state weekly measurements, and compared each measurement to the previous one, which revealed a reduction of circumference between each measurement in the intervention group, and between the first and last two measurements in the control group. The circumference reduced faster in the intervention group than in the control group. No intergroup comparison was performed. The authors conclude that kinesiotaping has a positive effect on ROM, quadriceps muscle strength, reduction of edema, and pain.

Balki et al. [14]included 30 male patients after anterior cruciate ligament (ACL) reconstruction in a randomized placebo-controlled trial. The 15 patients in the treatment group received a mixed lymph- and muscle-activating kinesiotaping, while the 15 patients in the control group received suprapatellar sham taping, also using kiensiotape The kinesiotape was applied twice, starting on day 4 after the intervention for a total of 10 days (i.e. change after 5 days of treatment), with measurements on postoperative day 8 and 13. The study investigated the effect of the kinesiotape on pain, swelling, range of motion (ROM), and muscular strength, and found consistent positive effects on swelling as well as night pain, ROM, and hamstring muscle strength at day 10 when comparing the two groups. The Lysholm and Tegner[48] score were comparable at 1 and 3 months postoperatively. The authors conclude a potential benefit on the above describes functions, which could positively influence rehabilitation programs after hamstring autograft-reconstruction of ACL injuries.

Sixty patients (30 per group) after ACL reconstruction were included in the study by Chan et al.[21] The intervention group was treated with kinesiotape in addition to standardized physiotherapy, the control group received standardized physiotherapy only. The treatment with kinesiotape started during the first postoperative week and lasted for 2 weeks. Outcomes of the study were pain, ROM, Lysholm-Tegner score[48], and the mid-patellar leg circumference. These aspects were performed in the early (first two weeks) and late (6 weeks) postoperative period. Data of the preoperative measurements were not reported in the article. Group averages of measurements were compared to the previous average values within the groups, and also between the groups. Intragroup comparisons revealed an improvement of all aspects except for early postoperative pain. Intergroup comparison revealed a stronger reduction in pain intensity in the intervention group at early, but not at late time points. The intergroup comparison revealed no significant difference for the Lysholm-Tegner score[43], ROM, or the mid patellar girth. The authors conclude that kinesiotaping had a positive effect on pain in the early postoperative phase after ACL reconstruction, but found no evidence for a benefit concerning the other analyzed aspects.

Donec et al.[17], investigated the effectiveness of kinesiotape application after total knee replacement in the early postoperative rehabilitation period in 89 patients who (40 intervention, 49 control group). All patients received standardized physiotherapy and psychosocial support, and the patients of the intervention group additionally received kinesiotaping from the second postoperative day after onwards for four weeks. The tape was applied every 7 days, with a day treatment pause between two applications. As outcomes, reduction of pain and edema as well as the improvement in the range of knee joint motion were analyzed. The circumferences of the leg were measured at four levels preoperatively and on postoperative days 2, 8, 16, 24, and 28. The data for the preoperative assessment were not reported. Mean values of measurements were analyzed as intra- and intergroup comparisons. For all outcomes except pain, the study found differences favouring the intervention at mid-term to late measurements with the expected significant intragroup changes during the course of healing. The authors conclude that kinesiotaping has a beneficial effect on pain, edema and knee extension in the early postoperative phase.

One additional study by Windisch et al. [11], by means of a prospective cohort design, compared the effect of kinesiotaping after total knee arthroplasty in an intervention group with data of a historical control group, i.e. a patient population with total knee arthroplasty that was previously treated at the same hospital and whose data was analyzed in the context of another study with the same parameters[38]. The control group was treated with a 24hrs arterio-venous Impulse System^TM^, the intervention group instead with kinesiotape applied on the first postoperative day. Additionally, both groups received standardized physiotherapy. As outcomes, leg circumference, wound secretion, and skin temperature were assessed daily during the first postoperative week. A significant difference was only observed for the temperature on the lateral aspect of the knee, with a higher temperature in the intervention group than in the historic control. All other aspects showed similar results over time in both groups, for which reason the authors conclude that there is no clinically relevant benefit of the kinesiotape application.

Gülenç et al. published two randomized controlled trials on the decongestive effect of kinesiotaping after arthroscopic surgery of the knee[20] and shoulder[22].

The first study included 42 patients with knee arthroscopy in an overnight setting[20]. They were evaluated at day 2, 8, 16, 24 and after 6 weeks for pain and leg circumference at four levels (above knee, knee. mid-calf, ankle). At the same time points taping was applied or renewed. 20 patients were treated with neuromuscular as well as decongestive kinesiotape application. One patient dropped out due to a skin irritation. The control group consisted of 21 patients treated with sham taping, which however was not described in more detail. The authors found a significantly lower postop. swelling aournd the knee in the kinesiotape group throughout the postoperative period, as well as a significantly reduced mid-calf circumference in the late postoperative course. There were no significant inter-group differences for pain or circumference at the other levels. The authors conclude that kinesiotaping had a positive effect on swelling after arthroscopy.

The second study evaluated 50 patients with arthroscopic shoulder surgery[22], of whom 24 were treated with kinesiotape in a decongestive technique and 26 as control group using sham taping with a seemingly paper-like standard tape. Taping was applied at the above mentioned time points. at which also measurements were performed. These evaluated the upper shoulder diameter between the posterosuperior acromion and the coracoid at the superior deltoid and a second diameter between the same reference points one cm laterally. In addition pain was assessed. There was a significant reduction in pain levels at the early postoperative time points and a significant reduction in mid-deltoid diameter between day 8 and 24 in the kinesiotape group compared to control. The authors conclude a predominant pain reducing effect in the early postoperative period.

**Maxillofacial Surgery**

Ristow et al.[16-18], published 3 studies on the subject of the effect of postoperative application of kinesiotape in maxillofacial surgery.

The first study[16], included 26 patients (with an equal group distribution) after open reduction and internal fixation of unilateral mandibular fractures. The intervention group received kinesiotaping in addition to analgesia and cooling, whereas the control group only received analgesia and cooling measures. The kiensiotape was applied immediately after surgery and left for at least 5 days. The primary outcome of the study was reduction of maximal swelling. Secondary endpoints were reduction of swelling at different time points, mouth opening, reduction of pain, and subjective outcomes concerning the treatment. The study revealed that the extent of maximal swelling was larger and appeared later in the control group than in the intervention group. Swelling regressed faster in the intervention group, so did the limitation of mouth opening. For pain, no significant difference was found between the groups. At early time points, patient satisfaction was slightly higher in the intervention group. The authors conclude that kinesiotaping is a “promising, simple, less traumatic, and economical approach for managing postoperative swelling that is free from systemic adverse reactions, thus improving patients’ quality of life.” [16]

In a second study[17], with each 20 patients per group after removal of all four wisdom teeth were evaluated with the same study design. The primary endpoint was the facial surface between postoperative days 0 and 2, and the authors used the same secondary endpoints as described above. Again, swelling peaked earlier, reached lesser extents, and regressed faster with the application of kinesiotape, which also coincided with better mouth-opening. Patients in the intervention group reported on average less pain and stated higher levels of satisfaction than the patients in the control group. The authors reached to the same conclusions as stated above, and further concluded that the application of a kinesiotape is associated with an improvement in postoperative morbidity, work capacity, and potential reduction in costs.

The third study[18c] included with the same design a total of 30 patients after zygomatico-orbital or zygomatic-maxillary fractures involving the orbital floor with indication for open reduction and internal fixation. The primary outcome was the increase in swelling during the postoperative course, which proved to peak and regress faster with kinesiotape treatment. Unlike in the other two evaluations, mouth opening and pain levels were not advantageous in the intervention group compared to the control group. The authors reached to the same conclusion as above, and state additionally that “even when swelling persists, kinesiotaping gives patients the impression of a minor swelling detracting them (*patients*) from their pain and morbidity.”

Tozzi et al. [15] included 24 patients (equally distributed) after bimaxillary orthognathic surgery with corticosteroid treatment as control and additional kinesiotape in the intervention group. The time point of the first application of kinesiotaping was not reported, and the kinesiotapewas left in place for 5 days. The outcomes of the study were swelling, pain, and mouth opening, as compared between the preoperative baseline and postoperative day 4. Swelling was assessed via the facial surface area with silicon masks that were analyzed using the Digitalizer 3-D^TM^. Concerning swelling, the study revealed more favorable results for the intervention group than for the control group, not however concerning pain and mouth opening. The authors conclude that application of kinesiotape is effective, not least due to the ease of application, low costs, and psychologic benefit for patients.
